# Effect of intravenous magnesium on post-operative pain following Latarjet shoulder reconstruction

**DOI:** 10.1177/17585732231158805

**Published:** 2023-02-27

**Authors:** Paul Soeding, Alex Morris, Adam Soeding, Gregory Hoy

**Affiliations:** 1 FANZCA Department of Anaesthesia and Pain Medicine, 90134 Royal Melbourne Hospital, Parkville, Australia; 2 Department of Pharmacology and Therapeutics, The University of Melbourne, Victoria, Australia; 3 The University of Melbourne, Victoria, Australia; 4 Department of Anaesthesia and Pain Medicine, 90134 Royal Melbourne Hospital, Parkville, Australia; 5 Department of Preventative Medicine, The Alfred Centre Monash University, Prahran, Australia; 6 Melbourne Orthopaedic Group, Monash University Department of Surgery, Windsor, Australia

**Keywords:** magnesium, Latarjet, pain relief, surgery, nerve block, rebound

## Abstract

**Background:**

Single injection ropivacaine interscalene anesthesia (ISA) is frequently used in Latarjet reconstruction to enhance post-operative analgesia. A potential limitation is the occurrence of severe rebound pain on block resolution. We investigated the effect of intravenous magnesium on post-operative pain, particularly at the transition of block resolution to multimodal analgesia.

**Methods:**

Elective patients (*n* = 40) having Latarjet open shoulder reconstruction were randomised to receive either intravenous magnesium sulphate 50 mg/kg (M) or normal saline (S) before induction. Post-operatively, a standardised analgesic regimen was used, and post-operative pain was recorded using a verbal numerical rating assessment (VNRA) score. Requirement for injected opioid analgesia was recorded.

**Results:**

ISA provided longstanding analgesia in all patients with block duration slightly prolonged in the magnesium group (16.7(1.0) (S), 17.8(1.3) h (M), *p* = 0.049). Magnesium resulted in less rebound pain following ISA resolution (VNRA 4.0 (0.6) M, 6.2 (0.8) S, *p* = 0.03) and lower pain intensity at 24 h. Four patients had nausea and two required rescue opioid injection.

**Conclusion:**

Magnesium before Latarjet surgery results in less rebound pain following ropivacaine block and improves post-operative analgesia. Magnesium may be indicated in major upper limb surgery, where significant pain intensity is anticipated.

**Level of evidence:**

Treatment study; Randomised blinded; Level 2.

## Introduction

Open Latarjet shoulder reconstruction may be associated with severe pain, and clinical experience indicates that postoperative pain can vary significantly between patients. Single-injection interscalene anesthesia (ISA) is frequently used since it provides complete anaesthesia of the shoulder joint and excellent post-operative analgesia.^1^ In addition, ISA facilitates rapid post-operative recovery, and with long-acting agents such as ropivacaine, patients experience prolonged comfort.^2,3^ However single injection techniques are limited by their duration of action. On block resolution, some patients may experience sudden rebound pain with significant distress, often necessitating longer admission times.^4^ Multi-modal analgesia may alleviate this pain, but in many patients, this transitional period remains poorly managed. Single-shot techniques are, therefore, viewed by some as having minimal post-operative benefit,^5,6^ and avoided unless a continuous infusion via catheter^7,8^ is used to prolong block duration. However, catheter techniques are not appropriate for many ambulatory procedures, and themselves have limitations.^1,9,10^

A number of studies have shown magnesium therapy to be effective in surgical patients, by decreasing both post-operative^11^ and chronic pain.^12^ Magnesium infusion before induction has been demonstrated to decrease post-operative pain scores, using either visual analogue scale (VAS) or verbal numerical rating assessment (VNRA). Opioid consumption is also decreased. The anti-nociceptive effect of magnesium is speculated to involve interaction with the spinal cord *N*-methyl-d-aspartate (NMDA) receptor, resulting in the attenuation of pain transmission, and prevention of central sensitisation.^13^ Magnesium may also inhibit peripheral sensitisation, via inhibition of calcium influx into nociceptor neurons.^14^

Our practice is in accordance with current guidelines,^15^ by administering multimodal analgesia pre-emptively^16^ before ISA resolution. For both arthroscopic and open shoulder surgery, this approach effectively controls the severity of rebound pain in most patients, but not all. The aim of this study was to investigate the effect of intravenous magnesium, administered before surgery, on post-operative pain. We hypothesised magnesium to decrease both post-operative and rebound pain score, and opioid consumption. Patients scheduled for similar surgery (Latarjet open reconstruction) were specifically chosen.

## Methods

Following Institutional Ethics review (ACTRN12617001320347), and informed written consent, 40 patients scheduled for elective Latarjet reconstruction were prospectively recruited. Patients were randomised, and investigators were blinded to treatment. Patients with a chronic pain syndrome, conduction defect or pacemaker, renal failure, hypocalcaemia, magnesium or calcium channel blocker treatment, or inability to communicate VAS scoring, either due to language or miscomprehension was excluded.

Pain was assessed using a VNRA score to categorise pain. Patients subjectively described pain on a scale of 0–10, instructed to rate pain intensity as none (0), mild (2–3), moderate (5–6), severe (7–8) or worst pain imaginable (9–10). Assessment was made at rest and on body movement. Specific time intervals monitored were 0–6, 6–12, 12–18, 18–24 and 24–30 h. For each time period, the maximum VNRA was recorded, and the effect of rescue analgesia was noted. Myles et al.^17^ have reported a minimal clinical important difference (MCID) in VAS of 10 mm is clinically significant and suggests a value of 33 represents acceptable pain after surgery.

Surgery involved a standardised delto-pectoral approach, using a subscapularis split, and performing the open Latarjet procedure using an orthograde (rather than congruent arc) coracoid bone graft fixed by two cannulated titanium screws and washers (Mitek, Rayneham, Mass, USA) performed by a single surgeon. The transferred tendons (short head of biceps and coracobrachialis) remain attached to the transferred bone to produce a sling effect on the joint post-operatively. The musculocutaneous nerve is routinely dissected and retracted. A drain is inserted post-operatively for 12–18 h.

On arrival at theatre, patients were randomised (via computer-generated table) to receive either magnesium (50 mg/kg in 50 ml) or saline infusion 30 min before induction. Infusions were then prepared and masked by a research assistant. Operating clinicians were blinded to randomisation and pain assessment. Only in the event of an adverse event would the clinician be informed of which treatment was administered.

Patients were monitored during infusion (ECG, SpO_2_, NIBP), after which they were then supinely positioned, and an ultrasound-guided ISA was performed. Ropivicaine (25–30 ml, 0.75% with dexamethasone 25 ug/ml) was injected into the interscalene space, adjacent to the C5 and C6 nerve roots. General anaesthesia was induced with propofol (15–20 mg/kg), with a laryngeal mask airway placed into the airway for controlled ventilation. Anaesthesia was maintained with sevoflurane (end-tidal concentration, Et_SEVO_ 1.5%). Non-invasive blood pressure, ECG, temperature and pulse oximetry were measured continuously. All patients received prophylactic intravenous cefazolin and ondansetron.

In the post-operative period, patients were assessed for the level of post-operative pain and need for analgesia. Of interest was the increase in pain during transition from regional anaesthesia to oral analgesia, the requirement for rescue opioid injection, and incidence of nausea or complication. A pre-emptive^16^ analgesic regime was commenced before block resolution – intravenous parecoxib (40 mg) given in post-anaesthetic care unit (PACU), followed by regular administration of slow-release oxycodone /naloxone (10/5 mg) and paracetamol, commenced at 12 h post ISA, or at the first indication of block resolution. For breakthrough pain, patients were administered instant-release oral oxycodone (5–10 mg), and if required, intramuscular morphine injection. Patients were questioned on the incidence of post-operative nausea, vomiting, quality of sleep as well as the occurrence of dyspnoea or neuropraxia. Enquiry was also made to patient's experience of ISA and anaesthesia, and at discharge overall satisfaction (%) with the anaesthetic approach.

Patients were admitted overnight and specifically assessed for the first onset of pain, pain intensity, analgesic requirement, the presence of nausea and overall satisfaction with perioperative care. Pain scores are assessed using a VAS diary (VNRA 1–10) at 6-hourly intervals starting from entry into PACU. The time of partial and complete ISA block resolution together with VNRA score is recorded. On the following day, pain at the time of discharge is recorded, and follow-up at 24–36 h is made via phone call. The need for medical review, return to clinic or local doctor to control pain after discharge, is specifically enquired for.

### Statistical analysis

Data are presented as mean (SD). A previous audit has shown ISA duration in similar patients to be 15.4 (2.5) h, and the VNRA score in patients with severe rebound pain to be 7.5 (SD 2.7). We estimate a 33% difference in VNRA score to be clinically measurable, and analysis using G Power indicates a sample number of *n* = 20 per group is required when using a Student t-test to compare groups.

## Results

The study recruited 40 patients of similar age and BMI (Table 1). General anaesthesia was similar in both groups with respect to propofol induction dose (S 151(65) mg, M 170 (27) mg, *p* = 0.22) and end-tidal sevoflurane concentration (1.6–1.7%). There was no difference in intraoperative heart rate or mean arterial blood pressure between groups. The duration of surgery was 51.7 (2.0) min in the saline group, and 52 (1.7) min in patients receiving magnesium. All patients had similar wound drainage, blood loss averaging 150 ml before removal.

**Table 1.** Patient characteristics and response to transitional analgesia.

	Saline	Magnesium	*p*
M/F	16/4	18/2	0.40
Age (years)	27.1 (11.3)	29.1 (10.0)	0.57
BMI (kg.m^−^2)	24.0 (1.7)	25.4 (2.7)	0.07
Surgery time (min)	51.7	52.0	0.9
Rescue morphine (*n*)	2	0	0.15
PONV (*n*)	4	2	0.40
Satisfaction (%)	90 (10)	91 (6)	0.65

BMI: body mass index; PONV: post-operative nausea and vomiting.

**Figure 1.**
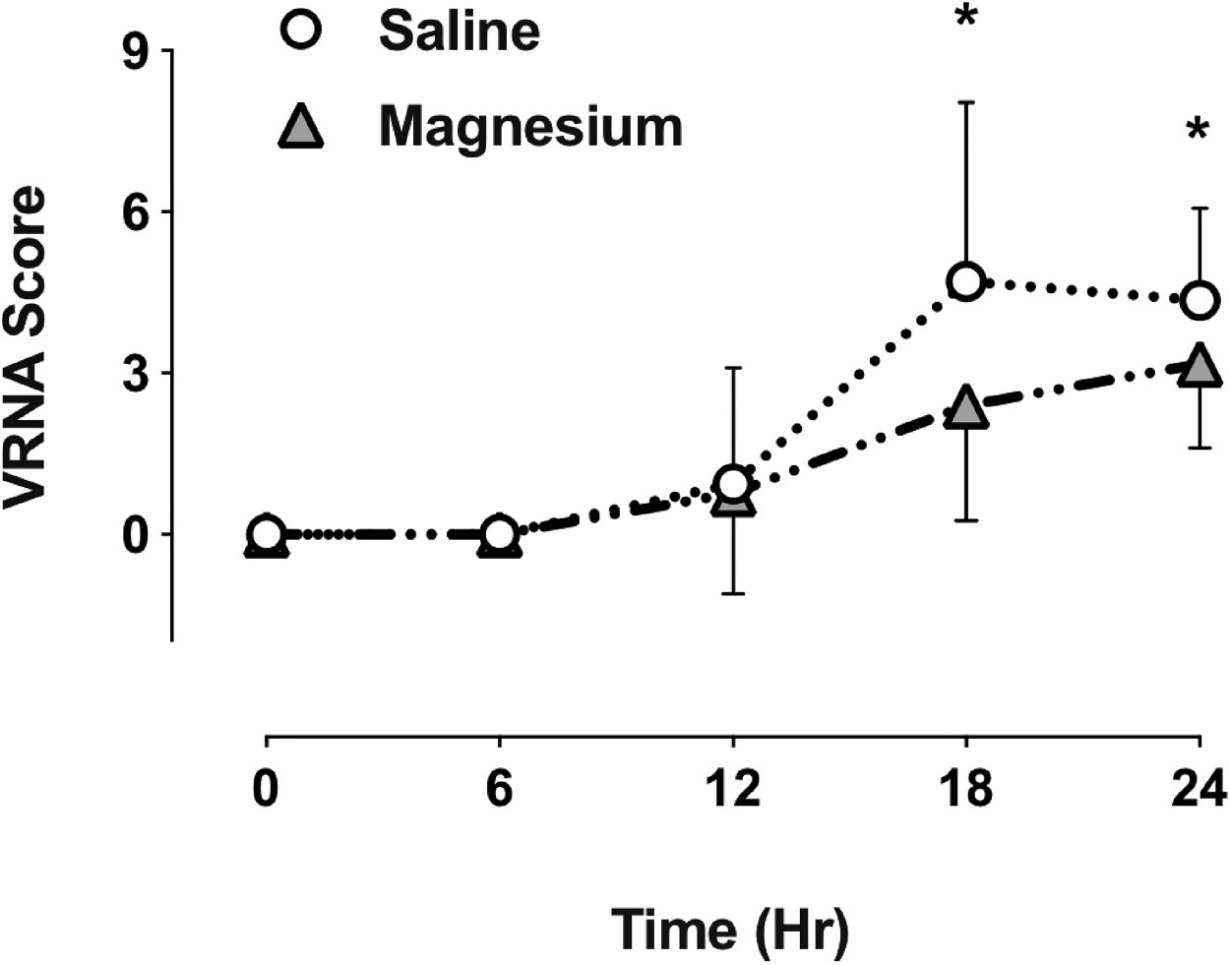
Post-operative pain intensity (VNRA score) following Laterjet open surgery. Patients received magnesium or saline infusion before induction. PACU time 0 h, discharge approx time 24 h. * p < 0.05.

**Figure 2.**
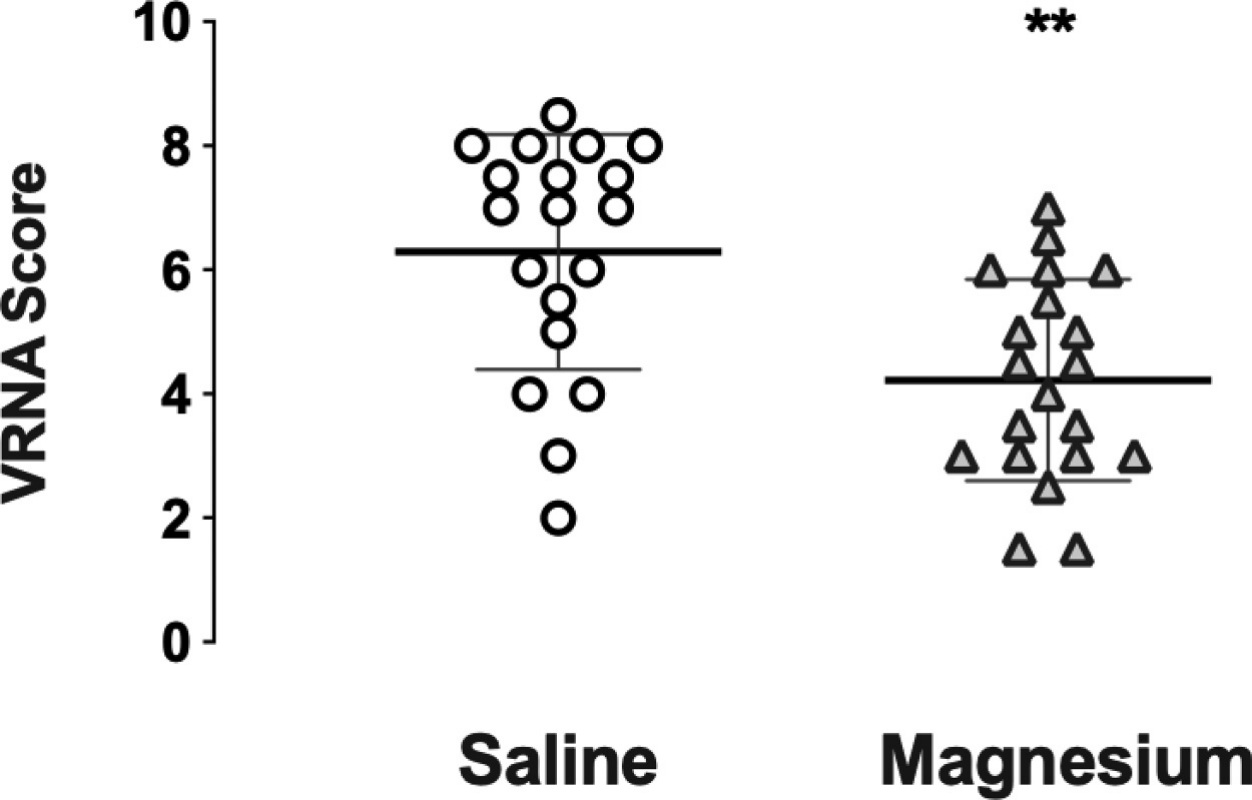
Maximum pain intensity (VNRA score) at time of interscalene block resolution, also termed “rebound” pain, following pre-emptive analgesia. Individual data plotted, showing group mean and dispersion of scores. ** p < 0.01.

**Figure 3. fig3-17585732231158805:**
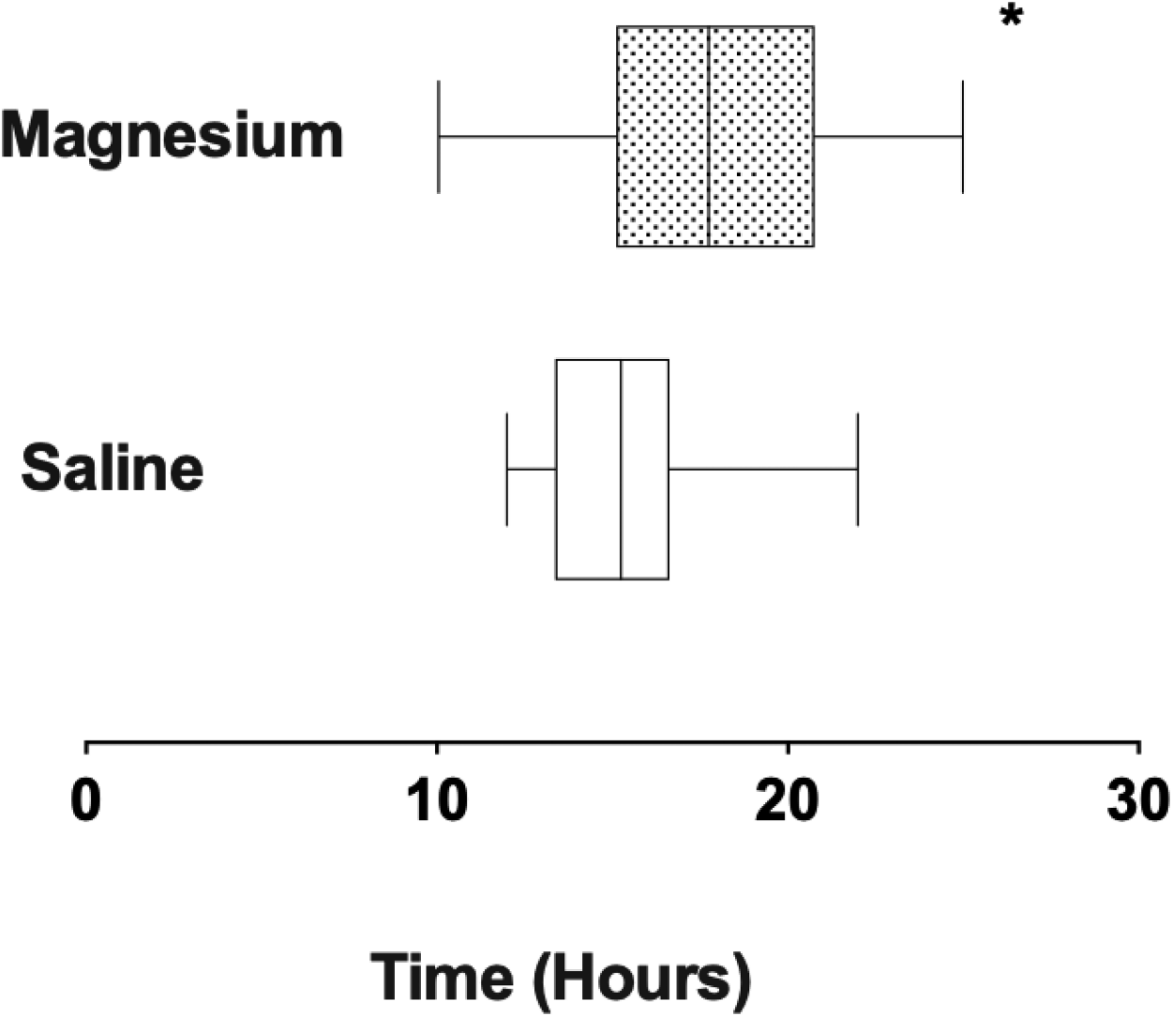
Post-operative duration of interscalene anesthesia in patients receiving magnesium or saline before induction. * p < 0.05.

In the PACU, patient recovery was rapid, without nausea or vomiting, and brachial plexus anesthesia was clinically evident in the operated limb (Figure 1). Patients were positioned with head elevation to 45°, the surgical arm held across the chest in a sling. On questioning all patients were comfortable, with VNRA score of 0, facilitating rapid return to the ward. ISA provided longstanding post-operative analgesia in all patients (Figure 2) with block duration being slightly prolonged in the magnesium group (Figure 3, mean duration S 16.7(1.0), M 17.8(1.3) h, *p* = 0.049).

A standardised oral analgesic regimen was given pre-emptively and regularly to all patients. With sling immobilisation numerical pain scores at rest and on movement (limited by sling) were reported as very similar in most patients, and when different, the higher VNRA score was recorded. Rebound VNRA on ISA resolution was significantly less in the magnesium group, 4.2 (0.6) versus 6.3 (0.8), *p *= 0.01. Most patients were comfortable with one or more doses of instant release oxycodone (in the 12-h interval between slow release) but in two control patients, rescue opioid injection (5–10 mg morphine) was required during this transition period for excess breakthrough pain. Apart from these two patients, there was no difference in opioid consumption between the groups. At 24 h most patients reported scores less than 5, with magnesium patients scoring significantly lower (S 4.4 (0.6), M 3.2 (0.7), *p* = 0.04).

On return to the ward, three patients expressed displeasure concerning the presence of an insensate arm. No patient complained of post-operative dyspnoea, related to diaphragmatic paresis, or experienced neuropraxia or seizure related to ISA insertion. Four patients experienced nausea from oral medication, and two patients in the saline group required rescue opioid, with one requiring a further day of hospital admission. At discharge patient satisfaction was high in both the saline and magnesium groups (90(10), 91(0.7)%, with all patients accepting a similar approach for future surgery.

## Discussion

This study has shown that intravenous magnesium given before Latarjet surgery results in less rebound pain during ISA resolution, and improvement of pain intensity following transition to oral medication. With ISA, all patients remained comfortable for at least 13 h post-operatively, a consistent finding when dexamethasone is added to ropivacaine.^18^ Patients treated with magnesium, however, had a modest prolongation of ISA, and on block resolution, VNRA scores were 2.1 lower than control patients. Following transition to multimodal analgesia, both groups reported acceptable levels of pain at 18–24 h, again with the magnesium group reporting a score of 1.2 lower at 24 h. These findings are in agreement with other studies, performed in patients receiving general anaesthesia alone^11^ – including obstetric,^19^ gynaecological,^20,21^ spinal^22^ and thoracic^23,24^ patients, with most reporting less post-operative pain, and a decrease in analgesic requirement with magnesium supplementation. In regional anaesthesia, one study reported a 20% increase in ISA block duration and decrease in pain scores at 12 h.^25^

The anti-nociceptive effect of magnesium demonstrated in our study and others is not surprising. Magnesium is postulated to have a direct anti-nociceptive action within the spinal cord, primarily through antagonism of the NMDA glutamate receptor within the dorsal horn.^13^ Within the NMDA receptor, magnesium inhibits calcium channel activation and requires displacement for glutamatergic excitatory signalling to occur.^26^ Magnesium is also postulated to interact with peripheral nociceptors, attenuating surrounding tissue inflammation and/or modulating calcium influx into these neurons.^14^

In our experience, single injection ISA provides immediate surgical anaesthesia of the shoulder, intraoperative haemodynamic stability, rapid recovery and a pain-free return to the ward. This is particularly beneficial in elderly patients where the quality of recovery is improved with decreased anaesthetic requirement. Criticism of single-shot ISA^5^ often fails to acknowledge aspects other than post-operative analgesia, particularly intraoperative anaesthetic management. These benefits of ISA are balanced against the patients’ experience of a prolonged and insensate limb, as well as the risk of rare complication, including neuropraxia or seizure.^27^

However, alternative approaches to ISA, both regional and tissue infiltration, are also used to control post-operative pain after shoulder surgery. Wound infiltration with either plain or liposomal local anesthetic, for example, can be used for pain management. And a recent report describes the use of liposomal bupivicaine in ISA.^28^ Our findings are in agreement with the use of magnesium with techniques other than ISA. However, intravenous magnesium during surgery may have itself risk, with toxicity being associated with prolongation of neuromuscular blockade,^29^ conduction block or asystole.^30^ Although these effects are likely dose-related, meta-analysis of multiple studies report magnesium to have a relatively high safety profile, with hypotension or bradycardia occurring infrequently.^11^ In our patients receiving magnesium, the incidence of perioperative bradycardia or hypotension was not increased. However, beach chair positioning requires special consideration, where the vasodilatory effect of magnesium could potentially increase the risk of postural hypotension.^31^ In this situation, caution is recommended, and the use of fluid loading and/or vasopressor therapy is possibly indicated.

The strength of this study design is the use of a standardised surgical procedure – open Latarjet reconstruction, involving moderate skin incision, limited tissue dissection and traction, bone harvesting and tendon transfer. With the degree of tissue trauma likely to be similar in all patients, the comparison of post-operative pain between treatment groups was felt to be strengthened. A number of study limitations exist, none least the timing of rebound pain and pain assessment. As ISA diminishes, early or pre-emptive administration of multimodal analgesia is vital in preventing patient discomfort and potentially central sensitisation.^16^ The analgesic regimen following ISA resolution did eventually provide most but not all patients, with a comfortable overnight stay and ward discharge the following day. All patients expressed a high level of satisfaction at discharge and consented to repeat ISA should future surgery be required.

Pain is a complex experience and the assessment of pain following surgery has limitations. The significant variation between individuals is not only determined by the type and extent of surgery, but also by the interpretation of the pain itself. The experience of surgical pain is multifactorial and reliant on previous pain experience, gender, culture, socio-economic status and importantly phenotype.^32^ Accurate pain assessment in our patients was subject to these same constraints, particularly in determining the point of maximal rebound pain during ISA resolution. Clinical assessment of limb motor and sensory function at this time was limited, with limb immobilisation and pain on movement following surgery.

A limitation of many measurements is identifying a clinically meaningful change in pain score, which indicates a pain level acceptable for patient comfort. In a study of post-operative pain using the 100 mm VAS, Myles et al. reported that a minimal clinically important difference (MCID) in VAS of 10 mm is clinically significant and suggests a value of 33 represents acceptable pain after surgery.^17^ Similar values have been specifically described for rotator cuff surgery, with MCID reported as 14 mm, and acceptable pain levels as 30 mm.^33^

Our experience indicates single-shot ISA with multimodal analgesia is an effective technique for Latarjet reconstructions. Further, the infusion of magnesium before surgery contributes to decreasing post-operative pain, particularly rebound pain that occurs with interscalene block resolution. Magnesium supplementation may, therefore, be a valuable adjunct in patients undergoing major shoulder surgery.

## Conclusion

Peri-operative magnesium infusion decreases post-operative pain in patients undergoing Laterjet shoulder reconstruction, particularly when ISA with ropivacaine diminishes.[Fig fig3-17585732231158805]
